# Draft Genome Sequences of Five Enterococcus faecium Isolates from Traditional Montenegrin Brine Cheese

**DOI:** 10.1128/MRA.00353-20

**Published:** 2020-05-07

**Authors:** Werner Ruppitsch, Andjela Nisic, Anna Stöger, Franz Allerberger, Aleksandra Martinovic

**Affiliations:** a Institute of Medical Microbiology and Hygiene, Austrian Agency for Health and Food Safety, Vienna, Austria; b Department of Biotechnology, University of Natural Resources and Life Sciences, Vienna, Austria; c Faculty of Food Technology, Food Safety, and Ecology, University of Donja Gorica, Podgorica, Montenegro; University of Rochester School of Medicine and Dentistry

## Abstract

Enterococcus faecium is a multifaceted bacterial species. It is part of the natural human microbiota, it grows in a variety of traditional foods, and emerging multiresistant clones are a leading cause of nosocomial infections. Here, we present draft genomes of five E. faecium isolates originating from traditional Montenegrin brine cheeses.

## ANNOUNCEMENT

Enterococci, belonging to the group of lactic acid bacteria, are often present in traditional food products (1). Due to the emergence of multidrug-resistant enterococci as a leading cause of nosocomial infections (2), only specific strains of Enterococcus faecium can be used as probiotics or feed additives (3). The genus *Enterococcus* has not obtained the status generally recognized as safe (GRAS) (4). E. faecium strains exhibiting probiotic and other beneficial potentials must be differentiated from pathogenic strains, as determined by the European Food Safety Authority (EFSA) (5, 6). Whole-genome sequencing (WGS) analysis of E. faecium strains has the potential to distinguish between safe and potentially harmful strains, thereby minimizing possible risks for consumers (7, 8).

Bacteria were isolated from white brine-ripened traditional cheese from Montenegro, as described previously (9, 10). Colonies morphologically suspected to be enterococci were subcultured for species identification by WGS.

The GeneMatrix bacterial and yeast genomic DNA purification kit (EURx, Gdańsk, Poland) was used for the isolation of genomic DNA from overnight cultures grown in M17 or MRS broth (HiMedia, India). The Nextera XT kit (Illumina, Inc., San Diego, CA, USA) was used for WGS library preparation, and paired-end sequencing (2 × 300 bp) was performed on a MiSeq system (Illumina) as described previously (11). Default parameters were used for all software unless otherwise specified. Raw reads were quality controlled using FastQC v0.11.9. Trimmomatic v0.36 (12) was used to remove adapter sequences and to trim the last 10 bp of each sequence, as well as sequences with a quality score of <20. Reads were assembled using SPAdes v3.11.1 (13). Contigs were filtered for a minimum coverage of 5× and a minimum length of 200 bp using SeqSphere+ software v6.0.0 (Ridom GmbH, Würzburg, Germany).

WGS of five E. faecium isolates generated 1,053,512 to 1,933,204 reads, with a mean coverage of 39× to 48×. The NCBI Prokaryotic Genome Annotation Pipeline identified 2,721 to 2,840 genes, 2,639 to 2,690 coding sequences, 168 to 192 pseudogenes, 14 to 19 rRNA genes, and 61 to 68 tRNA genes (Table 1). Species identification was done with a BLAST search against the NCBI 16S rRNA database (14), Mash distance analysis (15), and ribosomal multilocus sequence typing (rMLST) (16). All methods identified all isolates as E. faecium. Strains were characterized by MLST (17) and core genome MLST (cgMLST) using SeqSphere+ with default settings, as described (18). All isolates had >97% good cgMLST targets and were sequence type 1453 (ST1453) and the new cgMLST complex type 2909 (CT2909) (CT founder strain INF40 [sample no. 511450]) (https://www.cgmlst.org/ncs/schema/991893/searchstrain/?f=sampleid&v=511450). cgMLST-based comparisons with genomes available in GenBank revealed that Montenegrin isolates differed by a maximum of 4 alleles from each other and by 63 alleles from the closest relative in GenBank, strain UC7267 (ST1453) (BioProject accession no. PRJNA200656), which was isolated from Italian cheese in 2002. BAGEL4 (19) and antiSMASH 5.0 (20) revealed that all isolates carried genes of the bacteriocin biosynthetic gene clusters (i.e., type III polyketide synthase, the RiPP cluster, and enterolysin A). Analysis of genomes with ResFinder (21), PlasmidFinder (22), and VirulenceFinder (23) from the Center of Genomic Epidemiology revealed that all isolates carried the intrinsic resistance genes *msrC* and *aac(6′)-li* and had mutations in *pbp5* conferring resistance to ampicillin C, carried the plasmids rep1 and repUS15, and carried virulence factors *acm* and *efaAfm*.

**TABLE 1:** Characteristics and accession numbers of genomes of E. faecium isolates from Montenegrin brine cheese

Strain	Genome size (bp)	No. of reads	Total no. of genes	No. of RNAs	Avg coverage (×)	No. of contigs	Contig *N*_50_ (bp)	G+C content (%)	GenBank accession no.	SRA accession no.
INF9	2,667,018	1,133,000	2,728	89	41	282	29,445	38.2	JAAHCC000000000	SRR11111645
INF12	2,656,101	1,628,168	2,721	81	41	417	15,070	38.3	JAAHCE000000000	SRR11111643
INF29	2,772,412	1,748,532	2,778	88	47	621	14,675	38.7	JAAHCB000000000	SRR11111646
INF39	2,641,555	1,933,204	2,756	82	48	625	9,506	38.6	JAAHCD000000000	SRR11111644
INF40	2,727,633	1,053,512	2,840	88	39	287	43,961	38.3	JAAHCA000000000	SRR11111647

### Data availability.

The Enterococcus faecium whole-genome shotgun project has been deposited in DDBJ/ENA/GenBank under accession no. JAAHCA000000000 (INF40), JAAHCB000000000 (INF29), JAAHCC000000000 (INF9), JAAHCD000000000 (INF39), and JAAHCE000000000 (INF12). The versions described in this paper are the first versions (accession no. JAAHCA010000000 to JAAHCE010000000). The raw sequence reads have been deposited in the Sequence Read Archive (SRA) under accession no. SRR11111643 (INF12), SRR11111644 (INF39), SRR11111645 (INF9), SRR11111646 (INF29), and SRR11111647 (INF40).
